# Evaluation of feasibility and user acceptance of lateral-flow self-testing for viral illness in a residential treatment rehabilitation facility

**DOI:** 10.1186/s40352-022-00173-x

**Published:** 2022-02-26

**Authors:** Benjamin L. Sievers, James Klotzle, Tipu V. Khan

**Affiliations:** 1 J. Craig Venter Institute, La Jolla, CA USA; 2 Pitzer College, 1050 N Mills Ave, Claremont, CA 91711 USA; 3 Prototypes/HealthRight360, 845 East Arrow Highway, Pomona, CA 91767 USA

**Keywords:** COVID-19, SARS-CoV-2, Influenza, Rapid test, Lateral-flow test, Viral screening, Self-testing

## Abstract

**Background:**

The role of rapid testing has proven vital in reducing infection incidence in communities through swift identification and isolation of infected individuals. The COVID-19 pandemic has been particularly catastrophic for residential carceral and rehabilitation facilities that are high-risk settings for transmission of contagious diseases. Centralized provider-based viral testing employing conventional diagnostic techniques is labor-intensive and time-consuming. There is a marked unmet need for quick, inexpensive, and simple viral testing strategies. We hypothesized that rehabilitation residents could successfully test themselves employing inexpensive, disposable, antigen-based influenza lateral-flow tests and would be willing to self-isolate and self-report to health authorities if positive.

**Methods:**

We evaluated self-testing among 50 rehabilitation residents ages 18 and older in Pomona, California, where participants self-administered influenza lateral-flow diagnostic test (without specimen collection) with the goal of appropriately observing a control line and completed two brief written surveys on self-testing and COVID-19, one before self-administering the lateral-flow test and one after, to determine the overall feasibility of viral self-testing and to characterize attitudes comparing self-testing and provider-based testing.

**Findings:**

A total of 50 rehabilitation residents were enrolled in this study and all 50 conducted a lateral-flow test and answered the provided surveys. Among the participants, 96% (48 of 50) achieved a positive-control line from their lateral-flow test. Most participants, 83% (34 of 41) indicated that they would prefer to perform their own rapid test instead of having a health care provider administer the test. Notably, 98% (49 of 50) indicated that they would self-isolate if the lateral-flow test returned a positive indicator suggesting the presence of a viral infection and 96% (48 of 50) would report positive results to their corresponding public health department.

**Interpretation:**

Residents in a residential rehabilitation center were widely able to successfully self-administer standard lateral-flow antigen-based rapid diagnostic kits. Self-testing was strongly preferred over tests administered by a healthcare provider. Reassuringly, almost every resident indicated that they would report any positive test result to the health department and self-isolate accordingly. Self-testing offers a promising adjunct to centralized testing, potentially better enabling swift and effective management of life-threatening infectious outbreaks among those living in high-risk congregate living settings.

## Introduction

Viral illness can be catastrophic for nursing homes, residential rehabilitation centers, and prisons because these communities face an unusually elevated risk as airborne viruses spread efficiently through indoor spaces. During the 1918 influenza epidemic, in San Quentin prison in April and May of 1918 26% of the 1900 prisoners were infected, making this outbreak one of the main foci of the 1918 influenza pandemic (Awofeso, 2004). After SARS-CoV-2 was identified among 6 newly transferred individuals at a Wisconsin prison, 79.4% of incarcerated persons and 22.6% of staff members were infected with SARS-CoV-2 clustering in the same genetic lineage over just 2 months in late 2020 indicating that infection was transmitted from these early transfers (Hershow et al., 2021). Similarly, by mid-March 2020 at Riker’s Island main jail complex in New York City, more than 200 cases were diagnosed within the facility only 2 weeks after the first COVID-19 diagnosis at the facility (Hawks et al., 2020). By June 6th, 2020, the COVID-19 case rate for prisoners was 3251 per 100,000 incarcerated individuals and a death rate of 39 deaths per 100,000 prisoners, considerably higher than the US population COVID-19 death rate of 29 people per 100,000 (Saloner et al., 2020).

Current diagnostic testing strategies that employ quantitative polymerase chain reaction (qPCR) assays are certainly sensitive and accurate but are time-consuming and technically challenging and thus don’t easily scale to meet high throughput demands typically found in carceral and residential settings (Mina & Andersen, 2021). Under standard protocols, qPCR assays require a minimum of 3–4 h from sampling to evaluation and test results are typically provided from 24 to 72 h later (Döhla et al., 2020). These traditional testing modalities also require expensive laboratory equipment not available to community residential centers. Additionally, when outbreaks are occurring in the community, the testing and result timeline is often delayed due to a surge in samples which may lead to false-negative results (Zimmer, 2020).

Rapid self-testing using inexpensive lateral-flow tests could serve as an important adjunct to standard central testing as presumptive diagnosis of viral infections might be efficiently achieved without requiring additional healthcare supervision. Importantly, self-testing for a variety of analytes has been exceptionally successful. The first home pregnancy test was created in the 1970s, followed by robust uptake of self-testing for infectious diseases such as HIV (O’Farrell, 2009). Although the Abbott binaxNOW COVID-19 Ag Card (BinaxNow) test was recently approved by the FDA for self-testing, lateral-flow tests for influenza and streptococcal infections are typically performed by health care professionals in centralized facilities (FDA, 2021). In the context of a rapidly advancing global pandemic, we hypothesized that individuals were: 1) capable of successfully performing the assay steps required for lateral-flow self-testing for viral illness, 2) willing to self-isolate if positive for infection, and 3) willing to report positive results to public health authorities.

## Methods

### Study design

This study sought to determine whether individuals were able to conduct a lateral-flow diagnostic test achieving a positive-control line and to characterize general attitudes towards self-testing. The study was conducted at the Prototypes/HealthRight360Women’s Center, a residential rehabilitation facility in Pomona, California that cares for a range of individuals including mothers from the California Department of Corrections and Rehabilitation. A total of 50 rehabilitation residents ages 18 and older were recruited for this study through informational handouts and word-of-mouth at the Prototypes Rehabilitation Facility. Upon recruitment into the study, the volunteers received a test kit containing: 1) one OSOM® Ultra FLU A&B lateral-flow test cassette, 2) one sterile flocked collection swab, 3) one single-use reagent package, 4) a pre-test survey and post-test survey, 5) assay instructions and 6) a modest cash compensation ($5) for their time and effort. The participating individuals were asked to answer a brief questionnaire survey prior to and after conducting the lateral-flow test to characterize opinions and attitudes towards COVID-19 self-testing, willingness to isolate if a positive result was identified, and willingness to report positive findings to health authorities. The ability to conduct the test was measured through the achievement of a positive-control line in the lateral-flow test cassette. Participants were instructed to forgo self-collection of a nasal specimen.

### Ethics

This study was approved by both the Institutional Review Board at Pitzer College and HealthRIGHT 360’s IRB committee, project number: 2020-32R1. Pitzer College’s institutional review board’s review was carried out in accordance with the requirements of Part 46, “Protection of Human Subjects” of Title 45 of the Code of Federal Regulations and the “US Department of Health and Human Services (DHHS) Federal-Wide Assurance (FWA) for the Protection of Human Subjects for Domestic (US) Institutions,” Pitzer College Assurance #FWA00001138.

### Procedures

Following written consent, the participating rehabilitation residents were asked to complete a pre-test survey. After completing the pre-test survey, each participant was given the self-testing kit that contained an OSOM® Ultra Flu A&B lateral-flow test (Sekisui Diagnostics) which is Food and Drug Administration (FDA) approved and Clinical Laboratory Improvement Amendments (CLIA) waived. The participants were provided a visual demonstration of conducting the test created by the manufacturer and a stepwise description; these instructions included the amount of time for each step and proper collection techniques. Following the execution of the lateral-flow test, the participants then complete the post-test survey.

### Sample size, participants, and demographic data of the facility

**Fig. 1:**
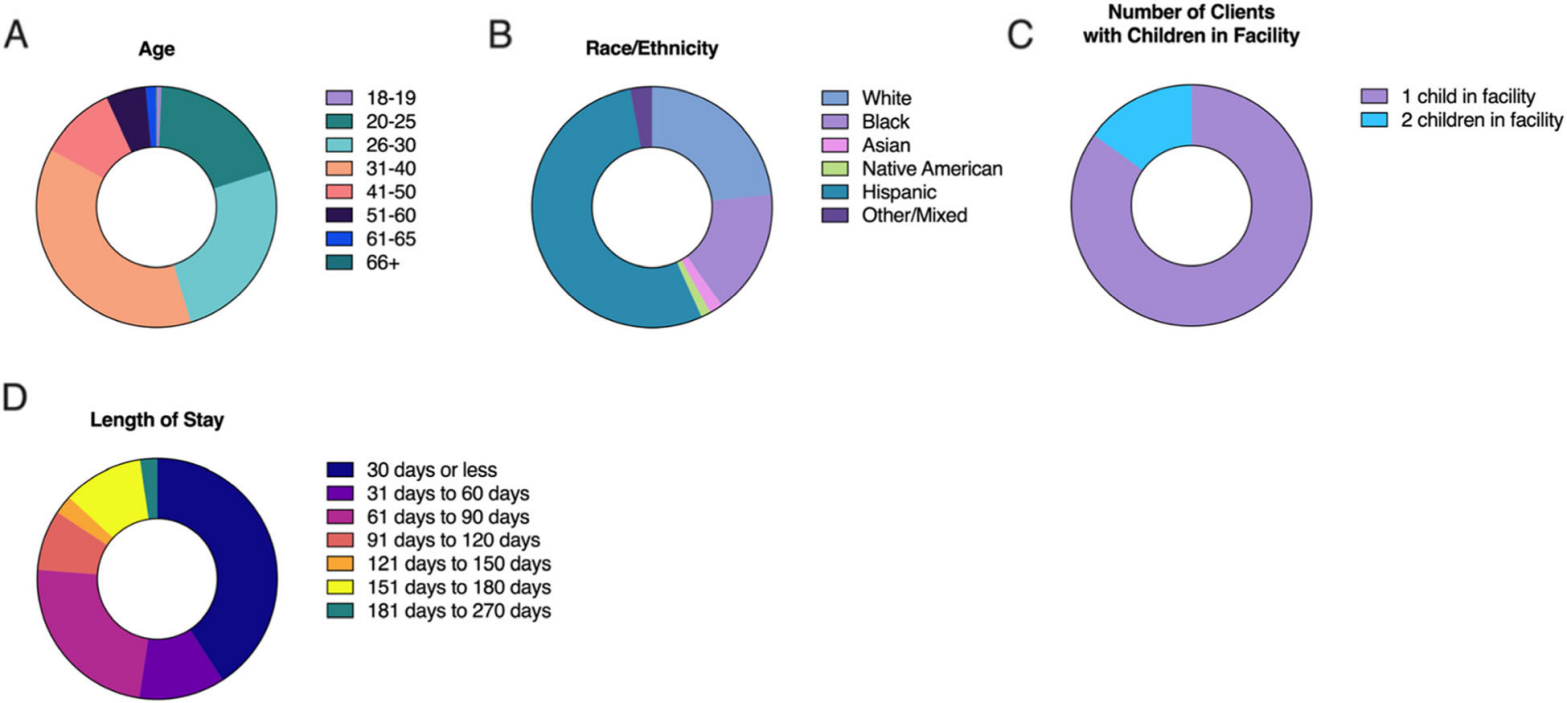
Demographics and additional characteristics of the 2020–2021 Prototypes rehabilitation population. **A** Age distribution, **B** Racial demographics, **C** Total number of clients with children, and **D** Distribution of the average length of stay

The sample size consisted of 50 rehabilitation residents 18 years of age and older living at the Prototypes Women’s Rehabilitation facility located in Pomona, California. General demographics of this relatively small facility are provided to help maintain the anonymity of individual participants (Fig. 1).

The residential program at Prototypes Women’s Rehabilitation facility treats 613 individuals with 96 active and 517 who exited from the program at the end of 2020. Of the 613 individuals at the facility 53.8% (330/613) are Hispanic/Latinx, 1.7% (11/613) are Asian, 16.8% (103/613) are Black, 1.3% (8/613) are Native American, and 23.3% (143/613) are White (for all purposes of CalOMS Hispanic/Latinx clients are counted as white in the race classification and Hispanic in the Ethnicity classification).

### Survey design

There were two metrics used to determine feasibility and attitudes towards self-testing: 1) the pre-test and post-test surveys and 2) whether or not a positive control line was produced. The method of data collection through pre-test and post-test surveys were used to quantitate the impacts, usability, and feasibility of the lateral-flow test experiment experienced by the participant. The surveys included 7 questions and provision of a relative rating score regarding a statement on a scale from 1 to 7 starting from strongly disagree (1) to strongly agree (7). At the bottom of each survey, there was an optional anonymous response section to provide any additional comments regarding their overall experience in a free text format.

### Role of the funding source

This study was funded by a grant awarded to Benjamin Sievers from the Pitzer College Racial Justice Initiative. Pitzer College had no role in study design, collection of data, or writing of the report. The corresponding author had full access to the data and ultimate responsibility for the decision to submit for publication.

## Results

### Survey answers measuring self-testing attitudes and opinions before conducting a self-test

**Fig. 2:**
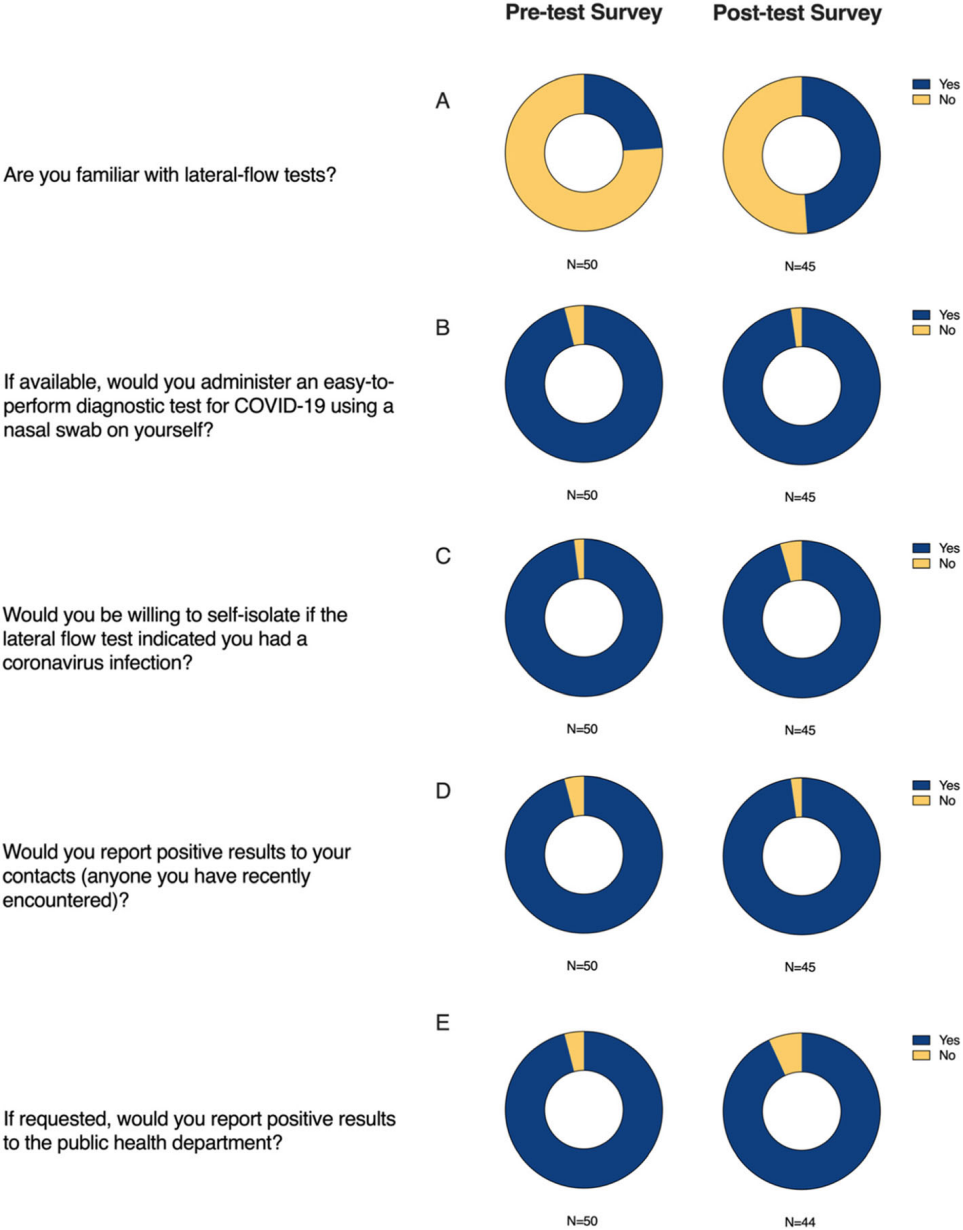
Overall distribution of answers to each of the questions asked on the pre-test and post-test surveys. The pre-test survey was taken before the self-administration of the lateral-flow test, likewise, the post-test survey was taken after completion of the lateral-flow test

Among 50 participating rehabilitation residents enrolled in the study 48 (96%) reported achievement of a positive control line on the OSOM® influenza lateral-flow test. Of the 41 participants that indicated their testing preferences, 34 of the 41 (83%) indicated that self-testing was preferred over healthcare administered testing. Nine participants did not answer this question. With regards to reporting positive results recorded from self-testing, 49 of 50 (98%) indicated that they would self-isolate after receiving a positive indicator of a viral illness. Likewise, 48 of the 50 (96%) indicated that they would report the positive results to the corresponding public health authority (Fig. 2).

Participation in the post-test survey was lower than the pre-test survey with 45 of 50 (90%) participants completing the post-test survey. Among the 45 participants that completed the post-test survey, the participants’ willingness to report positive results to a corresponding public health department slightly decreased to 41 of 44 (93.2%). Interest in conducting a COVID-19 diagnostic self-test increased to 44 of 45 (97.8%) participants agreeing that they would prefer to self-administer a COVID-19 nasal swab diagnostic test.

**Fig. 3:**
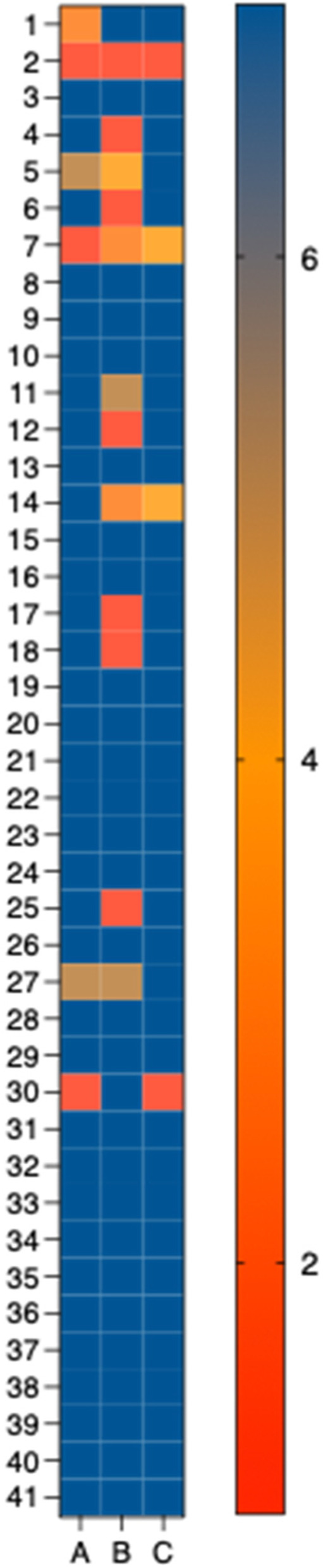
Heatmap of all individual participant responses employing a 7-point scale (1: strongly disagree to 7: strongly agree) that characterize opinions about self-testing in response to the following statements. **A** “I felt comfortable administering my own test,” (**B**) “the test was not difficult to perform,” and (**C**) “I could teach others to perform these tests.” The mean responses to questions A, B and C were 6.36 +/− 1.69, 5.61 +/− 2.35, and 6.56 +/− 1.43 respectively. Nine of 50 participants did not submit responses. The 7-point heatmap scale is provided to the right of the figure

Each post-test survey included three statements about the self-testing experience that had a seven-point rating scale (1: strongly disagree to 7: strongly agree). The first statement, “I felt comfortable administering my own test,” received an average response score of 6.37 with 35 of 41 participants giving the statement a 7 conferring strongly agreeing with the statement. For the second statement, “the test was not difficult to perform,” participant response varied with 7 of 41 participants reporting that they strongly disagreed and 29 of 41 strongly agreeing that the test was not difficult to perform. The second statement had an average response score of 5.61 out of 7 which is a lower average score than the other statements. The last statement, “I could teach others to perform these tests,” had an average response score of 6.56 out of 7 with 37 of 41 rating the statement a 7, strongly agreeing that they would be able to teach others to perform a self-test (Fig. 3).

### Reported experiences with self-testing

Before and after conducting the lateral-flow test a series of questions on the surveys were used to gauge attitudes and previous experiences with self-testing. Prior to conducting the lateral-flow test, each participant was asked their familiarity with lateral-flow tests. Of the 50 participants, only 12 of 50 (24%) were previously familiar with lateral-flow tests. After completion of the lateral-flow test, the number of participants that felt that they were familiar with lateral-flow tests increased to 22 out of 45 (48.9%). Although the majority of participants were largely unfamiliar with lateral-flow tests 96% were still able to conduct the test.

At the bottom of the survey, a small portion of the page was dedicated to providing feedback on the experience. One participant wrote, “the test was very easy to perform” and another participant stated, “either [testing strategies] work for me as long as the results are correct, but easier and preferably myself so it will be much faster and not have to be on a waiting list to test.” One participant stated, “it was awesome.” Another participant also noted that “it [self-testing] was a great idea.”

## Discussion

In this prospective evaluation of rapid lateral-flow viral-antigen self-testing in a residential rehabilitation setting, almost all rehabilitation residents (96%) reported successful achievement of a control line on their lateral-flow assay and the vast majority (83%) preferred the self-testing method over a healthcare-provider administered test. Additionally, 98% and 96% of participants stated that they would self-isolate and report their positive findings to health authorities after receiving a positive test result from a rapid self-test, respectively. Collectively, our findings suggest that lateral-flow-based self-testing is not only feasible but widely preferred by individuals over centralized testing performed by healthcare providers. As successful infection control in these settings requires relatively frequent surveillance testing, we believe that viral surveillance self-testing using inexpensive lateral-flow tests may more swiftly enable isolation of infected individuals to help prevent serious illness and deaths.

Reassuringly, our identification of a collective willingness of individuals to self-isolate and self-report newly-identified viral infection to health authorities suggests an accurate recognition of shared communal risks and reflects each individual’s commitment to the overall health of the residential community. Containment of contagious infections in congregate residential settings rely greatly on the willingness of infected individuals to self-isolate (Kucharski et al., 2020). In the collaborating facility for this study, positive rapid tests are followed up with a confirmatory PCR, separation, and a report to the relevant public health agencies for contract tracing. Coordinated rapid self-testing in congregate communities and populations that are at higher risk of worse health outcomes from COVID-19 and influenza, combined with willingness to self-isolate has the potential to protect against stealthy and harmful spread of the virus throughout communities (Rader et al., 2020).

Importantly, our study findings obtained in a residential treatment setting should not be broadly applied to all congregate carceral settings. For instance, it is not presently known whether a self-testing approach would result in a similar willingness to report and self-isolate in a maximum security facility. If positive findings from self-testing in more restrictive carceral settings mandate solitary confinement or adjacent isolation strategies, the self-testing strategy might have unintended negative consequences for infection containment.

While the United States is making significant strides making self-testing more accessible with the recent over-the-counter (OTC) approval of the BinaxNOW COVID-19 Ag Card and other various rapid COVID-19 home-tests, the majority of testing devices for viral infections still remain physician-administered or prescription only. Self-testing to detect infectious individuals before interacting with facilities (e.g., schools, airports, and restaurants), along with mask-wearing and adherence to other public health guidelines can be highly effective in suppressing transmission of SARS-CoV-2. Ideally, self-testing would be simple, inexpensive, and rapid, enabling an individual to screen themselves before interacting with others. There are manifold benefits to comprehensive use of self-testing to detect infectious individuals that include increasing an overall willingness to test and further engaging individuals to assume some autonomy of their healthcare (Katz et al., 2018).

HIV self-testing constitutes an ideal example of self-testing in action. In an online cross-sectional questionnaire from 2009, two-thirds of participants indicated that they would test more frequently if home HIV self-testing was available (Bavinton et al., 2013). In addition to providing rapid results, OTC HIV rapid tests for self-testing have been demonstrated to increase likelihood of testing in various formats, such as the ORAQUICK® HIV SELF-TEST (Orasure Technologies) (Pettifor et al., 2020).

Rapid self-testing is an excellent tool for combating a particularly contagious virus, providing results in minutes or hours rather than days often associated with centralized nucleic acid testing. Frequent self-testing uniquely prevents further viral spread into communities as it enables individuals to test regularly, increasing the chance of identifying asymptomatic cases which are responsible for a large proportion of SARS-CoV-2 infections (Oran & Topol, 2021). Widespread adoption of frequent rapid antigen self-testing in tandem with other infection prevention, mitigation, and identification strategies has the potential to prevent many COVID-19 deaths (Johnson-León et al., 2021).

Limitations and controversy surrounding self-testing rely on the fact that self-testing requires self-reporting of infectious cases, and importantly, self-isolation, however, self-testing for various health conditions including pregnancy and HIV have been shown to work successfully in the self-testing format (Wood et al., 2014; Lindner et al., 2021). Given a choice, rehabilitation residents markedly preferred self-testing to centralized provider-administered viral surveillance. Enabling rapid detection of infectious cases through self-testing is a powerful tool that can be used in future pandemics and should extend to more viruses than just SARS-CoV-2. Self-testing represents a powerful adjunct to current centralized testing strategies and holds considerable potential to save future lives.

## Conclusion

As residential carceral and rehabilitation communities are disproportionately affected by infectious diseases due to congregate settings, unique self-testing strategies are vital in preventing transmission of contagious diseases throughout these communities. Rapid self-testing has demonstrated to not only be possible as shown by one’s ability to complete a rapid test but preferred by a carceral community even increasing likelihood and frequency of viral testing. Residents in a residential rehabilitation center were widely able to successfully self-administer standard lateral-flow antigen-based rapid diagnostic kits. Self-testing was strongly preferred over viral tests administered by a healthcare provider. Encouragingly, nearly every resident indicated that they would report any positive viral test result to a corresponding health department and self-isolate appropriately. Self-testing provides a promising adjunct to centralized viral testing, potentially better enabling prompt and effective management of life-threatening infectious outbreaks among those living in high-risk congregate living settings.

## Data Availability

The datasets during and/or analysed during the current study available from the corresponding author on reasonable request.
